# Cardiac screening in adolescents: Insights from a 15-year Student Heart Check program

**DOI:** 10.1016/j.hroo.2026.03.006

**Published:** 2026-03-11

**Authors:** Tai G. Metzger, Matthew R. Cederman, Matthew Adams, Tyler S. Hershenhouse, Nolan S. Shoukri, Amr E. Abbas, Ahmad M. Suleiman, Simon R. Dixon, Jennifer A. Shea, Nishaki K. Mehta

**Affiliations:** 1 Oakland University William Beaumont School of Medicine, Rochester Hills, Michigan; 2 Cardiovascular Medicine, Corewell Health William Beaumont University Hospital, Royal Oak, Michigan

**Keywords:** Sudden cardiac death, Preventive cardiology, Electrocardiography, Echocardiography, Sports cardiology, Pediatric cardiology, Adolescent health

**Figure:**
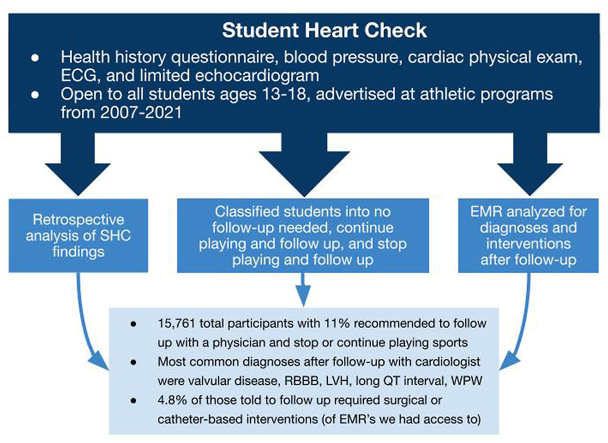

**Key Findings**

▪ Over a 15-year period, a comprehensive cardiac screening program including electrocardiography (ECG) and echocardiography evaluated 15,761 adolescents aged 13–18 years, identifying abnormal findings that warranted clinical follow-up in approximately 10% of participants.
▪ Those advised to stop sports participation showed significantly higher rates of syncope, ECG abnormalities, and structural heart disease.
▪ Among students with available follow-up data, clinically significant diagnoses included Wolff–Parkinson–White syndrome, long QT interval, left ventricular hypertrophy, valvular disease, and conduction abnormalities.
▪ The prevalence of serious conditions such as Wolff–Parkinson–White syndrome detected through screening was comparable with that reported in the general population, supporting the potential value of adolescent cardiac screening.

Sudden cardiac death (SCD) is a leading cause of nonaccidental death among young athletes.^1^ To prevent SCD, cardiovascular screening among young athletes has been supported by professional organizations, including the American Heart Association and the International Olympic Committee, but screening with an electrocardiogram (ECG) and echocardiogram has not been universally endorsed owing to a lack of randomized controlled trials.^2^ Screenings by pediatricians before sports participation often include personal history, family history, and physical examination (PE), but rarely ECG and echocardiogram. This study aimed to assess the impact of a cardiac screening program for adolescents from 2007 to 2021.

The Student Heart Check is a community-based program offering cardiac screenings consisting of a health history questionnaire, blood pressure (BP), cardiac PE, ECG, and limited 6-view echocardiogram. The screenings were available to all students aged 13–18 years at local schools and advertised to athletic programs. History and PE were based on the American Heart Association 14-point preparticipation evaluation and performed by an adult or pediatric cardiologist. Abnormal BP was considered greater than 120/80 mm Hg.^3^ ECG was interpreted using international criteria regardless of age or definition as an athlete. An adult or pediatric cardiologist interpreted the screening results and decided whether follow-up was advised.

We studied retrospective screening data and demographic data and diagnosis after follow-up with a pediatric cardiologist using electronic medical records for those with records available at Corewell Health. We did not classify participants into athletes and nonathletes. Data analysis was performed using REDCap and RStudio. Procedures were approved by the Corewell Health Institutional Review Board and adhered to relevant ethical guidelines outlined in the Declaration of Helsinki; a written consent was waived by the institutional review board owing to low study-associated risks and feasibility.

During the study period, 15,761 individuals were screened with characteristics: age 15.5 ± 1.5 years, 35% female, 80% Caucasian, 6% black, 3% Asian, 9% other race, and 2% undesignated. All students received echocardiograms after 2013, and only students with abnormal findings before 2013 (14,527 total).

Abnormal BP was identified in 2775 (18%). Other findings included history of dizziness/syncope (n = 423; 3%), abnormal PE (n = 1119; 7%), abnormal ECG (n = 1450; 9%), and abnormal echocardiogram (n = 284; 2%), with 1 or more abnormal findings leading to a recommendation for follow-up. In total, 1654 students (10%) were advised to follow up and continue playing, whereas 142 (1%) were advised to at least temporarily stop playing sports and follow up. Follow-up was with either a primary care physician or a pediatric cardiologist, whereas follow-up data were examined only for those who saw a pediatric cardiologist. The continue playing group had higher rates of abnormal BP (55% vs 23%), whereas the stop playing group had higher rates of syncope (13% vs 5%), abnormal ECG (53% vs 20%), and abnormal echocardiogram (32% vs 11%) (Figure 1A).

**Figure 1:**
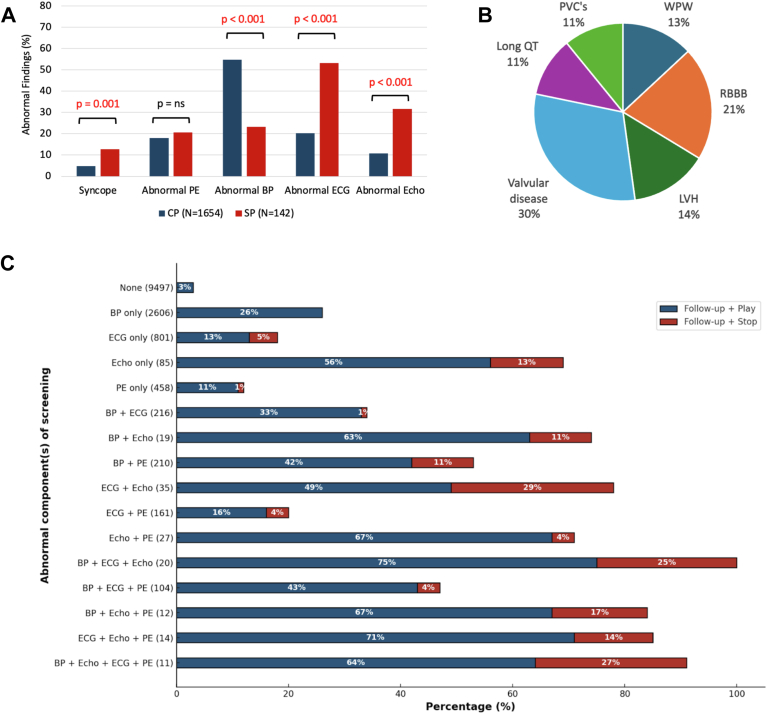
Abnormal findings and follow-up outcomes in the Student Heart Check program. **A:** Frequency of abnormal findings among students advised to CP vs those advised to SP. SP participants had significantly higher rates of abnormal ECG and echocardiographic findings (*P* < .001). **B:** Distribution of the most common diagnoses identified after cardiology follow-up, including valvular disease (30%), RBBB (21%), LVH (14%), WPW (13%), long QT interval (11%), and PVCs (11%). **C:** Follow-up recommendations based on combinations of abnormal screening components (BP, PE, ECG, and echocardiogram). Most participants with isolated BP abnormalities were cleared to CP, whereas those with multiple concurrent abnormalities had a higher likelihood of being restricted from play. Note: Data in panel C exclude blank responses for BP, PE, ECG, or echocardiographic abnormalities. BP = blood pressure; CP = continue playing; ECG = electrocardiogram; LVH = left ventricular hypertrophy; PE = physical examination; PVC = premature ventricular contraction; RBBB = right bundle branch block; SP = stop playing; WPW = Wolff–Parkinson–White syndrome.

We had access to the electronic medical record of 10% of the 1796 students (n = 188) advised to follow up. The most common final diagnoses after follow-up evaluation were valvular abnormalities (n = 28), complete or incomplete right bundle branch block (n = 19), left ventricular hypertrophy (n = 13), Wolff–Parkinson–White (WPW) (n = 12), premature ventricular contractions (n = 10), and long QT interval (n = 10) (Figure 1B). Less common findings included bicuspid aortic valve (n = 8) and atrial septal defects (n = 3). ECG abnormalities included WPW (n = 12), bradycardia (n = 9), rightward axis (n = 5), and first-degree heart block (n = 2). 9 students (4.8%) required surgical or catheter-based interventions, including atrial septal defect repair, cardiac myxoma removal, Ebstein’s anomaly repair, and catheter ablations for arrhythmia (all WPW except for 1 atrioventricular nodal reentrant tachycardia). There were no deaths noted for those who had follow-up evaluation with Corewell Health.

This retrospective analysis of 15 years of cardiac screening with the use of a health history questionnaire, BP, PE, ECG, and limited echocardiogram in adolescents by a single health system–based program demonstrates potential detection of significant cardiovascular abnormalities in this population, with 10% of students being recommended to follow up with a physician. Our results suggest that universal screenings with ECG and echocardiograms may detect some conditions at a comparable or higher rate than previously reported.^4^ For example, WPW has a prevalence of 1–3 per 1000, whereas we found 12 cases of 15,761, excluding cases diagnosed at other institutions.^5^ Although some diagnoses after follow-up (eg, incomplete right bundle branch block) can be acceptable according to international criteria, the participants were advised to follow up owing to other findings. In addition, only 60 students were told to follow up with an abnormal echocardiogram while having normal PE, BP, and ECG, indicating that more research may be needed to determine the role of echocardiograms as a screening tool.

The limitations include a lack of follow-up data for 90%, being a single-program analysis, a lack of generalizability owing to limited diversity and focus on athletes, and application of the ECG international criteria without determination of athlete status. Despite these limitations, this study revealed that cardiac screening identified individuals with cardiac abnormalities, which, in some cases, needed direct treatment. Future studies should continue to evaluate the effectiveness of cardiac screening based on diagnostic outcomes rather than prevention of SCD alone.

## Disclosures

The authors have no conflicts of interest to disclose.
